# Supplementary material for: Regulation of Ebola GP conformation and membrane binding by the chemical environment of the late endosome
Source: PLoS Pathog. 2023 Dec 6;19(12):e1011848. doi: 10.1371/journal.ppat.1011848 (PMC10727438; doi:10.1371/journal.ppat.1011848)
Supplement: Attachment — Submitted filename: Rebuttal to reviewers comments.pdf [file ppat.1011848.s005.pdf]

Response to Reviewers

We greatly appreciate the time that the Reviewers spent evaluating our work. We have carefully considered all their thoughtful and informed comments during the extensive revision of the manuscript. These revisions include significant new experiments and simulations. As a result of the Reviewers' analysis, we feel that the manuscript is now more complete and focused. Here, we respond to all the Reviewers' comments and outline the revisions.

<u>Part I – Summary</u>

Reviewer 1

Comment**:** This manuscript addresses mechanistic questions surrounding the processes of ebola virus glycoprotein mediated membrane fusion. The manuscript is predicated on significant prior work in the field that has indicated an importance for low pH to drive entry (though there is some controversy over this, as there is at least one report arguing that pH is not critical). More recently, calcium has also emerged as a potential cofactor for fusion peptide function. The authors here undertake a biophysical analysis of EBOV GP entry. The authors use an elegant biophysical approach to look at the interaction of a truncated EBOV GP, establish an assay with liposomes that optimize interactions of the truncated GP, and probe the impact of calcium and pH. The authors also use alanine scanning approaches to re-investigate GP mutants made in prior studies of viral entry and function as well as testing some new mutants based on hypotheses surrounding acid-mediated entry. The strength of this manuscript was the development of a biophysical approach to investigate the association of GP with liposomes and the use of FRET to look at changes in structure. The ability to then investigate the changes in association of the non-mutated GP with different membrane compositions was seen as strong. Beyond this finding, this reviewer struggled with the additional approaches and interpretation. Some of this difficulty came through the writing, which at times was incredibly dense and challenging to follow (e.g. there are single paragraphs that go on for a page, there are several conclusions drawn that seem to have little data backing). Other issues included a desire to have the results here explained more fully in the context of the significant prior art around EBOV GP virus fusion and entry to provide more understanding of what aspects of study presented her are unique against a background extensive other work. The study of point mutations to understand function could have been significantly more rigorous. While alanine scanning mutations are a good initial approach for defining important residues, for the additional depth of conclusion that the authors would like to make, less disruptive mutations (e.g. changing acidic residues to polar residues, etc) seem important and this approach has been applied effectively by others studying EBOV GP. While the use of MD to generate hypotheses is an interesting idea that is part of this manuscript, the lack of testing of hypotheses generated made conclusions that the MD was completely correct seem premature.

Response: **We thank the Reviewer for their supportive comments regarding our biophysical methodology. We also acknowledge that the previous version of this manuscript lacked readability at points and was incompletely integrated into the context of prior work. The manuscript has been largely rewritten with the intention of clarifying how we feel that our work makes advances beyond what has previously been published. In short, important prior**

work on EBOV GP-membrane interaction has focused mainly on the isolated fusion loop, and the identification of critical residues therein. Here, we focus on intramolecular (and inter-protomer) interactions that mediate the conformation of trimeric GP and its responsiveness to acidic pH and Ca^{2+}.

We are also in complete agreement that the previous version of the manuscript did not integrate our MD simulations adequately, nor were the predictions made by the simulations explicitly tested. We have reorganized the manuscript and expanded upon the mutagenesis to properly test the predictions made by the simulations (see below for more detail).

Reviewer 2

Comment: This is an interesting study on the pH and calcium dependence of Ebolavirus glycoprotein, considering both conformational changes and membrane binding. Overall, I think this is a valuable addition to the literature. There are two areas in which the work is weaker, which I highlight below for improvement or alteration.

Response: **We thank the Reviewer for their supportive and thoughtful comments. We have addressed these comments below pertaining to PS dependence and the rigor of our MD simulations. We feel that the manuscript is much stronger because of these additions.**

Reviewer 3

Comment: The manuscript submitted to PLOS Pathogen by Jain et al. investigates Ebola virus (EBOV) fusion initiated by its surface protein GP in the late endosomal compartment. In more detail, the authors assessed the impact of the endosomal biochemical environment such as pH, Ca2+ and membrane lipid composition on GP's conformational changes leading to fusion loop engagement of target membranes. They especially highlight the lipids phosphatidylserine and bis(monoacylglycero)phosphate enriched in late endosomal membranes as essential for GP-membrane interaction dependent on low pH and Ca2+ levels. They then identified residues within the GP fusion loop interacting with Ca2+ leading to local conformation changes and GP residues sensing low pH which releases the fusion loop and makes it available for membrane insertion.

Overall, the manuscript is well written, a more thorough investigation of the described biochemical environmental factors is of interest to the filovirus field and the experiments presented seem well executed. Nevertheless it is my opinion that the manuscript is lacking important controls as well as statistics and the authors should address the following questions:

Response: **We thank the Reviewer for their positive feedback. We also acknowledge that the previous version of the manuscript was lacking in several respects, which we have diligently addressed below.**

Part II – Major Issues

Reviewer 1

Comment: A main thrust of much of the manuscript is the proposed importance of the H154 residue for acid sensing. The H154A mutant examined here was earlier reported to have stability and VLP incorporation issues in an earlier report (Manicassamay) and these stability and infectivity issues are also seen here. The structural issues make it difficult to distinguish misfolding from loss of pH sensitivity. To make the conclusions that the authors propose, it seems important to identify a point mutation that maintains GP stability and now no longer shows pH dependent association with lipids. Recent work has provided examples of point mutations that stabilize the stability of GP so the identification of mutants that don't destabilize the structure but do remove the pH dependent conformational change does not seem to be an unreasonable approach for rigor.

Response: **We agree that the Manicassamy et al. (JVI 2005) work is critical to consider in interpretation of our work. Like Manicassamy et al., we observed reduced expression and pseudovirion incorporation of GP H154A. However, unlike Manicassamy et al., we evaluated the structure and conformation of H154A in multiple ways. First, we used an ELISA with the neutralizing antibody KZ52 to probe native prefusion conformation of trimeric GP. KZ52 requires GP1 and GP2 to be in a proper pre-fusion conformation for binding (J. E. Lee et al., 2008). The results from our ELISA show that binding of KZ52 to H154A mutant is comparable to wild type indicating a near-native pre-fusion conformation. Second, FCS is exquisitely sensitive to aggregation of the fluorescent species. We would expect a protein with a folding defect to show signs of aggregation in FCS. However, we observed no signs of aggregation of H154A as indicated by its diffusion time, 0.28 ± 0.02 ms, which is comparable to wild-type GP (0.308 ± 0.007 ms). Finally, our smFRET experiments indicated the same four FRET states for H154A as for wild-type GP. We would expect a misfolded protein to show a highly heterogeneous FRET signal, lacking well-defined states. We are thus confident that the loss of pH sensitivity seen in our experiments does not arise due to structural defects, and we have noted this in the revised Results. In contrast, Manicassamy et al. did nothing to evaluate the structure or conformation of H154A. They only tested expression using denaturing gel electrophoresis and pseudovirion infectivity. In their case, the lack of infectivity likely reflected the fact that no H154A GP was present on the pseudovirion.**

We would also like to point out that in the revised manuscript we have introduced two additional mutations (H154K and E178D) aimed at testing our predictions about the role of H154 protonation and its interaction with E178 and Y534 during fusion loop release. We feel these new data strengthen our story and we appreciate the Reviewer prompting us to think more deeply about our claims.

Comment: It seems that only selective effort has been made to link the findings here to prior work. There has been careful analysis of mutations within the fusion loop through both pseudovirus and lipid mixing assays that offer different strengths and weaknesses to the approaches here. It would be helpful to address this work so that the efforts here are more easily appreciated in terms of prior art.

Response: **We acknowledge that the previous version was insufficiently discussed in the context of prior studies. In this revision, we have been diligent to better identify how our work builds upon that of others. We have made special effort in the Discussion section to align our work with previous studies of GP fusion loop residues (e.g. Lee et al., PLoS One 2016; Nathan et al. ACS Chem Biol 2020).**

Comment: The authors re-examine an alanine mutation of histidine 516 and conclude it is an important residue for acid-based entry. Their conclusion (that its protonation is important for entry and therefore a factor in acid-dependent fusion/entry) is in contrast to other work that used a much more extensive point mutation approach and came to the opposite conclusion. The authors do not address this discrepancy nor do they explore the mutational landscape in the same depth that the prior work did. It is challenging to agree with the hypothesis put forward in this manuscript with out an effective rebuttal of the prior work.

Response: **We acknowledge that the previous version of this manuscript did not clearly indicate how our conclusion about the role of H516 is distinct from a prior study. Lee et al. (PLoS One 2016) demonstrated that a positive charge at position 516 was insufficient to promote lipid mixing mediated by the isolated GP fusion loop. We do not dispute this. Our conclusion is that H516 mediates conformational changes of *trimeric GP* and contributes to positioning the fusion loop for interaction with the membrane. The work of Lee et al. on the isolated GP fusion loop did not take into consideration the role of other amino acids that could interact with the fusion loop or the allosteric interactions observed in the GP trimer that might regulate GP conformational changes. Therefore, we felt that a re-examination of H516 in the context of the native GP trimer was warranted. Notably, our conclusion and the conclusion of Lee et al. are not mutually exclusive.**

The only point at which we diverge from Lee at al. is regarding the infectivity of the H516A mutant; we observe virtually undetectable infectivity in our lentiviral pseudovirion assay, whereas Lee et al. found no loss of virus entry using EBOV VLPs. We feel this is likely due to the reduction in expression of H516A, which probably lowered the density of GP on our lentiviral particles to the point that no infectivity could be detected.

We also acknowledge that in the previous version we did not use mutagenesis as comprehensively as Lee et al. In this revision, we have expanded our mutational landscape to explicitly test our model for the role of H516 and its interaction with W104 (H516R, H516W, and W104E). And now included in the Results, we show that the presence of a positive charge or an aromatic residue at position 516 supports the GP conformational change necessary for membrane binding.

Comment: MD simulations can be important for providing mechanistic insight into interactions that have been carefully probed. the application of the modeling in this instance suggests a mechanism but this mechanism is not tested through mutagenesis and should be if they are to be incorporated into the final manuscript.

Response: **We are in complete agreement with the Reviewer that validation of MD simulations is important to provide mechanistic insights. As noted above, we have expanded our**

consideration of GP mutants to explicitly test the predictions made by our MD simulations. We have also reorganized the manuscript to emphasize how the simulations made specific hypotheses that are tested experimentally.

Reviewer 2

Comment: The authors use a membrane composition "designed to mimic the late endosome (PC:PS:BMP:Ch 40:40:15:5)". The mole fractions of components here are somewhat unusual, particularly in that phosphatidylserine is extremely high. I would refer the authors to the work of Kobayashi and Gruenberg (which indeed the authors cite elsewhere), which lists late endosomal phospholipid composition (excluding the cholesterol) as 48-51 mol % PC, 12-20 mol % PE, 15-16 mol % BMP, 6-9 mol % SM, and only 2-5 mol % PS. Even plasma membrane doesn't have as high PS as used here. The high PS mole fraction is of particular importance here because calcium can interact with PS, so it may affect the results. I would suggest the authors either cite specific data showing this high a PS mole fraction in late endosomes or repeat critical data with a lower mole fraction PS.

Response**: First, we thank the Reviewer for this informed comment, which prompted us to more carefully consider our membrane-binding assay and the way in which we describe it. We acknowledge that we were incorrect in our statement in the previous version of the manuscript that the membrane content was "designed to mimic the late endosome". Indeed, the Reviewer is correct that we have used a high (non-physiological) concentration of PS in our study.**

For the revised manuscript, we included a PS titration in our FCS assay to indicate that 2-5mol% PS led to limited dynamic range in our detection of GP-membrane binding. Raising the PS concentration boosted membrane binding and increased the dynamic range of the assay. This enabled testing GP residues that mediate conformational changes necessary for membrane binding. Additionally—and importantly—the elevated PS did not alter the pH- or Ca^{2+}-dependence of the GP-membrane interaction. We therefore propose that BMP is the critical determinant of the Ca^{2+} dependence. In summary, the elevated PS was a tool employed in our in-vitro assay that enabled robust detection of GP-membrane binding and was not meant to emulate the late endosome. We presume that additional factors or components of the late endosome, which have not yet been identified, enhance GP-membrane binding in vivo at the physiological PS concentration.

Comment: Secondly, the molecular dynamics simulations presented in the manuscript do not meet current standards in the field for sampling and convergence. 225 ns seems a bit arbitrary as a simulation length, but more importantly multiple simulations should be run for each condition to obtain a better-converged estimator of the desired observables. Peter Coveney and colleagues have written extensively about this in terms of "aleatoric uncertainty". If this substantial expansion of the scope of simulations proves a burden to the authors, I might suggest that the manuscript stands very well without the MD simulations and they could be omitted entirely while still having a very strong paper.

***Response*: Here again, we thank the reviewer for the informed comment, consideration of which has strengthened our manuscript. Although it was not clear in the initial version of the manuscript, the MD simulations were a critical hypothesis generating exercise. In order to make this clearer, we have followed the Reviewer's guidance and expanded upon the simulations. Both simulations—with protonated or unprotonated histidines—have now been conducted in triplicate for 250 ns, generating an aggregate of 750 ns per protomer in the GP trimer. Although not called for by the Reviewer we have also conducted constant-pH MD simulations (in triplicate) to generate more accurate estimates of the pKas for the histidines of interest. Finally, we feel that the reorganization of the manuscript now more clearly indicates the importance of the MD simulations in driving our study.**

Reviewer 3

Comment: Do the Ca2+ concentrations used in the FCS and FRET experiments reflect concentrations that can be measured in endosomal compartments? The authors should comment on the question if the applied Ca2+ concentrations correlate with physiological endosomal Ca2+ concentrations and how potential differences impact the author's conclusions.

***Response*: The Reviewer raises an important point, to which we have given careful consideration. Estimates of endosomal Ca^{2+} concentration vary widely and likely depend on cell type, growth condition, and the method of measurement. Admittedly, the Ca^{2+} concentration used in our in vitro assays (1 mM) is likely higher than the concentration in the endosome. We have taken reference from previous work where the GP-membrane interaction has been studied at Ca^{2+} concentrations ranging from 0.5 to 2 mM. We opted to maintain this precedence to ensure compatibility with previous measurements. The results of our FCS and smFRET assays are overall consistent with the behavior of GP mutants observed in infectivity assays. Furthermore, one of our key conclusions is that BMP promotes GP^{CL}-membrane binding, which, according to our results, occurs across the physiological range of Ca^{2+} concentrations. We are therefore confident that the concentrations of Ca^{2+} in our assays are not influencing our conclusions.**

Comment: While the authors speculate briefly on the role of the receptor NPC1 for EBOV fusion, they have not introduced NPC1 (or NPC1-loopC) in their experiments especially the FCS assays. Especially in Figure 2, binding of GP to liposomes is modulated by its lipid composition and Ca2+ concentration - NPC1 should be included in this assay for example by employing liposomes decorated with loop C. It has been shown in previous publications that NPC1 is essential for fusion and infection to occur and the author's should make a larger effort to include NPC1 while discussing their work.

***Response*: We steadfastly agree that a mechanistic understanding of the role of NPC1 is of critical importance and ongoing work in our lab is devoted precisely to this issue. The goal of the present study is to identify the chemical constituents of the endosome that promote GP conformational changes such that initial contact between GP and the membrane is enabled. We believe, based on substantial prior studies of EBOV and other viruses, that this interaction**

is mediated by the fusion loop of GP. The conformational changes in GP that expose the fusion loop are therefore our focus here. Coating the liposome with NPC1 would lead to GP binding the liposome by way of its interaction with NPC1. We would therefore have no way to disentangle the GP-NPC1 interaction from the GP-membrane interaction, which is the focus of our present study. Ongoing work in the lab is seeking to elucidate the relationship between NPC1 binding and the mechanism of fusion triggering, which is a downstream event. We therefore prefer to address NPC1 in a later manuscript.

Comment: In Figure 3, differences between certain conditions (e.g. +/- Ca2+) are described as leading to a 'minimal increase' or 'increase to a greater extent' in GP-membrane interaction, but these differences are not evident from the bar graphs presented. For example, the text claims that cleavage of GP results in higher membrane binding at pH 5.5 in comparison to binding of uncleaved GP, but the difference seems to be minimal. Statistics supporting the author's claims should be included. Statistics are also needed for Figure 6A.

Response**: The Reviewer is of course correct in this assertion. We were remiss to not include statistical analysis in the first submission. We have included such an analysis throughout the revised manuscript, including in our presentation of FCS and smFRET data. FRET state occupancies are now presented as violin plots, which indicate the full breadth of the population, and permits statistical significance testing.**

Comment: The authors describe in the introduction that a point mutation at residue GP_H516 (suggested as a pH sensor) reduced fusion in vitro but this result could not be corroborated with cell-based fusion assays. Based on this prior observation, it would be essential to confirm in vitro results from FCS assays using GP with mutations at positions H139, H154 or H516 by cell-based fusion assays.

Response**: Indeed, as the Reviewer points out, a previous publication (Leet et al., PLoS One 2016) measured lipid mixing mediated by the isolated GP fusion loop (ie, a peptide) and found that the H516A mutation abrogated lipid mixing. At the same time, they found that the same mutation in the context of full-length trimeric GP had no effect on the entry of EBOV virus-like particles (VLPs) into cells. The explanation for this apparent discrepancy is unclear to us, except to say that lipid mixing mediated by the isolated fusion loop may not fully capture the complexity of membrane fusion mediated by the intact GP trimer. In the revised version of our study, we present data reporting on membrane-binding (FCS), GP conformation (smFRET), pseudovirion infectivity, and virion incorporation for all mutants under consideration. We find that these approaches yield overall consistent results.**

Comment: In Figure 4 the authors assess the impact of low pH and Ca2+ on GPCL binding to liposomes measuring conformational changes by FRET. In Figure 4D they show incremental decreases in GP pre-fusion conformations by introducing low pH, Ca2+, and membranes into the experiment. FRET data at pH 5.5 + liposomes (lacking Ca2+) should be added to further investigate the role of Ca2+ on GP's conformational changes.

Response**: The Reviewer is clearly correct that the role of Ca^{2+} in mediating GP conformational changes is of outstanding interest. In the revised manuscript, we present a more detailed view**

of our smFRET data, including indication of statistical significance of differences in FRET state occupancies. *Based on this analysis, Ca^{2+} has no detectable impact on the global conformation of GP.* We find these data to be conclusive, at least with respect to the conformational changes elucidated by the present smFRET assay. As discussed in the revised manuscript, we consider it likely that the role of Ca^{2+} is to mediate the local conformation of the fusion loop such that insertion into the target membrane is promoted. Unfortunately, we do not presently have an experimental means to elucidate these putative local dynamics. Although, MD simulations of the fusion loop in complex with Ca^{2+} are currently ongoing and will be reported in a future manuscript.

We have found that smFRET experiments, especially with a highly complex sample like a whole virus, are most interpretable when performed under conditions that minimize sample heterogeneity. Our FCS data, consistent with a previous report (Nathan et al., ACS Chem Biol 2019), indicate that Ca^{2+} is critical to maximizing GP-membrane interaction. smFRET data acquired in the presence of liposomes, but in the absence of Ca^{2+}, would yield a FRET distribution that is intermediate between the distributions seen at pH 5.5 with or without Ca^{2+} (which are nearly identical), and that seen in the presence of liposomes at pH 5.5 with Ca^{2+}. This distribution would only reflect the heterogeneity in GP-membrane binding. We would not be able to interpret that data in terms of the impact of Ca^{2+} on GP conformation; rather, we could only interpret it in terms of a loss in GP-membrane interaction. We therefore feel that that experiment would not report on the dynamics of interest.

Part III – Minor Issues

Reviewer 1

Comment: In this study, authors find positive effects of lipids and cation cofactors in association of wt GP with liposomes. Prior studies have found no effect using whole cell assays such as cell-cell fusion and pseudovirion assays that would investigate membrane fusion in contexts where lipids could be concave rather than convex as they are in liposomes. It would be important to discuss why the authors think that there is a difference in observed effects on GP entry using the different systems.

***Response*: We appreciate the Reviewer's insights on this important aspect of our study. Based on several prior studies of other viral entry pathways, we anticipated that composition of the target membranes would play a critical role. A previous study utilized assays for cell-cell fusion and pseudovirion fusion at the plasma membrane to explore the role of BMP (Feneant et al., PLoS One 2019). The authors concluded that BMP plays no role in EBOV fusion. However, in these assays, BMP was added to the cell culture media coupled with delipidated BSA. Delipidated BSA has been reported to improve the solubility of associated lipids and transport them into the cell lumen (Alsabeeh et al., BBA 2018). This method therefore does not offer control over the concentration of BMP available for interaction between GP and the target membrane. Also, plasma membranes have overall distinct lipid content from endosomes, which gives rise to distinct curvature, fluidity, and charge distribution. In our system, we use liposomes that are composed of lipids that are found in the late endosomes such as BMP and**

PS. The use of liposomes offers greater control over the concentration of lipids used and their availability for interaction with GP. This discussion is now included in the revised manuscript.

We would also like to point out that the Feneant et al. study was focused on triggering fusion, whereas we focused on GP-membrane binding. It remains possible that while BMP and PS are critical for the GP-membrane interaction, they are less critical for downstream fusion events. This is also now noted in the Discussion.

Comment: The authors make significant claims about 522 mutations and their importance for association and (by extension from pseudotype assays) fusion. Other reports have indicated only minor effects of the mutation on lipid mixing based fusion approaches. It will be important to address these differences and why the authors believe that their assay adds critical information that is not already present in the literature.

Response**: We thank the Reviewer for the informed input. To our knowledge, there are two reports that study the role of D522 in GP-membrane interaction and fusion (J. Lee et al., 2016; Nathan et al., 2020). Both reports focus on the role of D522 during lipid mixing mediated by the isolated fusion loop. The latter study also presents pseudovirion infectivity data exploring the role of D522. In the present work, we report on the role of Ca^{2+} and the D522 residue in specifically mediating the interaction between the *GP trimer* and a target membrane, and in mediating the conformation of the *intact GP trimer*. This information does not exist elsewhere in the literature. In the revised manuscript, this distinction between our study and prior work is made clear.**

Comment: The discussion does a great deal of re-summarizing data already summarized in the results and underemphasizes prior work done on EBOV entry.

Response: **We acknowledge that the previous version of the manuscript was lacking in its contextualization of new data in previous work. We have been diligent to address this shortcoming in the rewritten manuscript.**

Reviewer 2

Comment: The authors state that the H154A mutant was "insensitive to acidification, with a modest increase in transitions to low FRET" -- this seems a bit confusing as it would suggest modest sensitivity to acidification. Perhaps the authors could rephrase to clarify?

Response**: The description of the H154A mutant has been rewritten entirely to improve clarity.**

Comment: Since the authors report experiments both at physiological and nonphysiological calcium concentrations, perhaps a brief discussion of this would be helpful to readers.

Response**: In the revised Discussion section we have now included some context (and justification) for selection of our Ca^{2+} concentration.**

Reviewer 3

Comment: In the text authors are comparing their data in Figure 5A and 6A with wild-type data - to allow an easier comparison between data sets, wild-type data should be included.

Response**: The revised manuscript has been reorganized and data are now grouped according to mutant, with both FCS and smFRET data presented together for each GP mutant. To avoid repeating the complete wild-type data sets in 4 different figures (Figs. 2, 5-7), we have included wild-type data in the violin plots that display FRET state occupancies in each figure along with measures of statistical significance.**
